# Antiproliferative effect of boldine on neural progenitor cells and on glioblastoma cells

**DOI:** 10.3389/fnins.2023.1211467

**Published:** 2023-08-16

**Authors:** Enrique Jiménez-Madrona, Camilo J. Morado-Díaz, Rocío Talaverón, Arantxa Tabernero, Angel M. Pastor, Juan C. Sáez, Esperanza R. Matarredona

**Affiliations:** ^1^Departamento de Fisiología, Facultad de Biología, Universidad de Sevilla, Seville, Spain; ^2^Instituto de Neurociencias de Castilla y León (INCYL), Universidad de Salamanca, Salamanca, Spain; ^3^Departamento de Bioquímica y Biología Molecular, Facultad de Farmacia, Universidad de Sevilla, Seville, Spain; ^4^Instituto de Neurociencia, Centro Interdisciplinario de Neurociencias de Valparaíso, Facultad de Ciencias, Universidad de Valparaíso, Valparaíso, Chile

**Keywords:** hemichannels, neural stem cells, glioblastoma, connexins, pannexins

## Abstract

**Introduction:**

The subventricular zone (SVZ) is a brain region that contains neural stem cells and progenitor cells (NSCs/NPCs) from which new neurons and glial cells are formed during adulthood in mammals. Recent data indicate that SVZ NSCs are the cell type that acquires the initial tumorigenic mutation in glioblastoma (GBM), the most aggressive form of malignant glioma. NSCs/NPCs of the SVZ present hemichannel activity whose function has not yet been fully elucidated. In this work, we aimed to analyze whether hemichannel-mediated communication affects proliferation of SVZ NPCs and GBM cells.

**Methods and Results:**

For that purpose, we used boldine, an alkaloid derived from the boldo tree (*Peumus boldus*), that inhibits connexin and pannexin hemichannels, but without affecting gap junctional communication. Boldine treatment (50 μM) of rat SVZ NPCs grown as neurospheres effectively inhibited dye uptake through hemichannels and induced a significant reduction in neurosphere diameter and in bromodeoxyuridine (BrdU) incorporation. However, the differentiation pattern was not modified by the treatment. Experiments with specific blockers for hemichannels formed by connexin subunits (D4) or pannexin 1 (probenecid) revealed that probenecid, but not D4, produced a decrease in BrdU incorporation similar to that obtained with boldine. These results suggest that inhibition of pannexin 1 hemichannels could be partially responsible for the antiproliferative effect of boldine on SVZ NPCs. Analysis of the effect of boldine (25–600 μM) on different types of primary human GBM cells (GBM59, GBM96, and U87-MG) showed a concentration-dependent decrease in GBM cell growth. Boldine treatment also induced a significant inhibition of hemichannel activity in GBM cells.

**Discussion:**

Altogether, we provide evidence of an antimitotic action of boldine in SVZ NPCs and in GBM cells which may be due, at least in part, to its hemichannel blocking function. These results could be of relevance for future possible strategies in GBM aimed to suppress the proliferation of mutated NSCs or glioma stem cells that might remain in the brain after tumor resection.

## 1. Introduction

The mammal brain contains two regions in which neural stem cells (NSCs) persist after birth: the dentate gyrus of the hippocampus and the subventricular zone (SVZ), lining the walls of the lateral ventricles. In rodents, SVZ NSCs generate neural progenitor cells (NPCs) in asymmetric divisions which, in turn, divide to give rise to neuroblasts (Doetsch et al., 1997; Mirzadeh et al., 2008) that migrate toward the olfactory bulb and generate new neurons (Gage, 2000; Alvarez-Buylla and García-Verdugo, 2002). NSCs of the SVZ can also form oligodendrocyte precursors that contribute to the maintenance of the oligodendrocyte population in neighboring regions (Menn et al., 2006; Gonzalez-Perez et al., 2009; Capilla-Gonzalez et al., 2014). In humans, NSCs also remain in the postnatal and adult SVZ, although they are mainly committed to the oligodendrogenic process (Quiñones-Hinojosa et al., 2006; Capilla-Gonzalez et al., 2015). Compelling evidence from the last decade suggests that NSCs of the adult SVZ may be the cell of origin that encloses the driver mutations of glioblastoma (GBM), the most malignant primary brain tumor in humans (Lee et al., 2018; Matarredona and Pastor, 2019). Current treatment for GBM includes maximal surgical resection followed by radiotherapy and chemotherapy with temozolomide (Stupp et al., 2005). However, relapses occur, and patients have a median overall survival rate of 14–15 months (Thakkar et al., 2014). A better understanding of the biology of SVZ NSCs/NPCs will allow a deeper understanding of the etiology of this devastating cancer as well as the search for alternative therapies aimed at attacking the population of tumor-initiating cells.

NSCs/NPCs of the postnatal rodent SVZ can be isolated and amplified *in vitro* as floating cellular aggregates known as neurospheres (Reynolds and Weiss, 1992; Carpenter et al., 1999; Vescovi et al., 1999). Neurosphere-derived NPCs present hemichannels (formed by either connexin or pannexin subunits) that are open under control conditions (Wicki-Stordeur and Swayne, 2012; Talaverón et al., 2015). Connexin hemichannels allow the passage of ions and small molecules such as second messengers (inositol 3-phosphate, cAMP), and others (glucose, amino acids, nucleotides, ATP, NAD^+^) (Giaume et al., 2021). Communication mediated by connexin hemichannels is involved in the regulation of survival, proliferation, migration, oxidative stress, and gene expression in different cell types (Burra and Jiang, 2011). However, its precise role in postnatal neurogenesis has not yet been described. Among the 21 different connexin isoforms that form the hemichannels, connexin 43 (Cx43, 43 kDa) is the most abundantly expressed in the CNS (Contreras et al., 2004). The pannexin family includes three members, of which only the Panx1 and Panx2 isoforms are expressed in the CNS (Bruzzone et al., 2003). Panx1 hemichannel permeability is preferentially selective for anions (e.g., Cl^−^), and anionic small molecules (Ma et al., 2012), but the most studied intracellular metabolite released across the Panx1 channel is ATP (Yeung et al., 2020). Wicki-Stordeur et al. demonstrated *in vitro* that Panx1 hemichannels intervene in controlling NPC proliferation (Wicki-Stordeur et al., 2012) while *in vivo*, they promote NPC maintenance probably by facilitating the release of ATP, which could act as a survival (“do not-eat-me”) signal for resident phagocytic NPCs (Wicki-Stordeur et al., 2016).

NPC’s connexin hemichannels can also assemble and form gap junctions *in vitro* and in the postnatal SVZ neurogenic niche (Lacar et al., 2011). The relevance of this functional connectivity is still being studied. To date, some specific functions of gap junctional communication in SVZ-derived NPCs have been reported such as improved survival (Ravella et al., 2015), maintenance of the cellular architecture of the SVZ niche (Wiencken-Barger et al., 2007), or direct communication with blood vessels (Lacar et al., 2011).

Communication mediated by hemichannels is also relevant in the progression of GBM. Hitomi et al. proposed that gap junctions present in GBM and in glioma stem cells are essential for GBM growth, although the pro-tumorogenic role depends on the composition of the connexin subunits (Hitomi et al., 2015). Regarding pannexin hemichannels, *in vitro* experiments showed that Panx1 silencing inhibits the proliferation of U87-MG malignant glioma cells (Wei et al., 2015).

In this article, we aimed to evaluate the effects caused by the blockade of hemichannels on proliferation and differentiation of NPCs, isolated from the SVZ of postnatal rats. For that purpose, we used boldine, an aporphine alkaloid abundantly found in the leaves and bark of the boldo tree (*Peumus boldus*), that inhibits hemichannels formed by connexins and pannexins without affecting gap junctional communication (Yi et al., 2017; Cea et al., 2020). Our results show that proliferation of neurosphere-derived NPCs is reduced after treatment with boldine and that the inhibition of Panx1 hemichannels might be involved in this antiproliferative effect.

As SVZ NPCs might be the cell of origin of GBM, we have also explored the effect of boldine on established primary human GBM cells, showing that boldine treatment significantly reduces their growth. Our results suggest a key role for hemichannel-mediated communication in the mechanisms of glioblastoma progression and highlight boldine as an interesting compound to consider in GBM research or treatment.

## 2. Materials and methods

### 2.1. Neural precursor cell culture

Experiments were carried out on cells isolated from 7-day postnatal Wistar rats (P7) (*Rattus norvegicus*) of either sex according to the guidelines of the European Union (2010/63/EU) and Spanish law (R.D. 53/2013 BOE 34/11370–420, 2013) for the use and care of laboratory animals. Experimental procedures used in this study were approved by the ethics committee of the Universidad de Sevilla.

NPCs were isolated from the SVZ of P7 rats and were expanded in the form of neurospheres. Four P7 rats were used for each independent culture. Briefly, the lateral walls of the lateral ventricles were removed and enzymatically dissociated with 1 mg/mL trypsin (Invitrogen, Thermo Fisher Scientific, Waltham, MA, USA) at 37°C for 15 min. The tissue was then centrifuged at 150 g for 5 min, rinsed in Dulbecco’s modified Eagle’s medium/F12 medium 1:1 (DF-12; Invitrogen) and centrifuged again in the same conditions. Then, the cells were resuspended in DF-12 containing 0.7 mg/mL ovomucoid (Sigma-Aldrich, St Louis, MO, USA), and mechanically disaggregated by gentle trituration through a fire-polished Pasteur pipette. The dissociated cells were centrifuged and resuspended in defined medium (DM: DF-12 containing B-27 supplement, 2 mM Glutamax®, 100 units/mL penicillin, 100 μg/mL streptomycin and 0.25 μg/mL amphotericin B, all from Invitrogen) supplemented with 20 ng/mL epidermal growth factor (EGF; PeproTech, Rocky Hill, NJ, USA) and 10 ng/mL basic fibroblast growth factor (FGF-2; Millipore, Temecula, CA, USA). The cell suspension was maintained in an atmosphere of 5% CO_2_, at 37°C. After 1–2 days, cell aggregates named neurospheres were formed. Neurosphere cells were subcultured every 3–4 days by centrifugation, mechanical dissociation, and resuspension with fresh medium. All the experiments were performed with cells between passages 2 and 6.

### 2.2. Glioblastoma cell culture

Patient-derived primary GBM cells (GBM59 and GBM96 cells; kindly donated by Dr. Manuel Sarmiento, University of Seville) and U87-MG cells (ATCC, HTB-14™) were grown and maintained in DF-12 supplemented with 10% fetal bovine serum (Invitrogen), 2 mM Glutamax®, 100 units/mL penicillin, 100 μg/mL streptomycin and 0.25 μg/mL amphotericin B, all from Invitrogen.

### 2.3. Identification of hemichannel proteins by immunocytochemistry

Neurospheres-derived cells or GBM cells were seeded on 12-mm diameter coverslips in DF-12 with 1% fetal calf serum for 4 h to facilitate adhesion, at a density of 10,000 cells/coverslip (for neurosphere-derived cells, coverslips were pretreated with poly-L-ornithine, Sigma Aldrich). The coverslips were then fixed with 4% paraformaldehyde in 0.1 M phosphate buffer (10 min incubation). After several washings with phosphate-buffered saline (PBS), coverslips were incubated for 30 min in a blocking solution containing 2.5% bovine serum albumin in PBS and then 2 h at room temperature in a solution containing the primary antibodies (goat anti-nestin antibody, Novus Biologicals AF2736, 1:100, to identify neural stem cells, together with rabbit anti-Cx43 antibody, Thermo Scientific 71–0700, 1:200, or rabbit anti-Panx1 antibody, Sigma Aldrich HPA016930, 1:500). Then, coverslips were rinsed again and incubated for 30 min at room temperature with the corresponding secondary antibodies prepared in blocking solution (anti-goat IgG coupled to TRITC and anti-rabbit IgG coupled to FITC, Jackson ImmunoResearch, West Grove, PA, USA, 1:200). After washing, cells were counterstained with 4′-6′-diamidino-2-phenylindole (DAPI, Sigma-Aldrich, 0.1 μg/mL) for 10 min, washed again and mounted on slides with a n-propyl-gallate solution (Sigma-Aldrich, 0.1 M) prepared in glycerol:PBS 9:1.

### 2.4. Ethidium bromide uptake

Experiments were carried out in 24-well plates seeded with neurosphere-derived NPCs at a density of 10,000 viable cells/cm^2^ in DM, or in 24-well plates containing 12-mm coverslips seeded with GBM cells (5,000 cells/cm^2^) in GBM medium. Seventy-two hours after seeding, the medium was replaced by a HEPES buffered salt solution containing: 140 mM NaCl, 5.4 mM KCl, 1.8 mM CaCl_2_, 1 mM MgCl_2_, 10 mM glucose and 5 mM HEPES; pH 7.4, and incubated for 5 min. Then, cells were treated for 15 min with boldine (50, 100 or 300 μM, connexin and pannexin hemichannel inhibitor, hydrochloride form of boldine, kindly provided by Härting S.A., Chile), or with vehicle (MilliQ® water, control). Subsequently, ethidium bromide (EtBr, 5 μM) was added to the treated and control plates. Following a 10-min incubation with the dye, cells were washed with HEPES buffer, and coverslips were fixed with paraformaldehyde 4% in phosphate-buffered saline 0.1 M pH 7.4 for 10 min. For neurospheres, six photographs of each plate were taken with a Nikon inverted fluorescence microscope connected to a digital video camera (Leica DC100). For GBM cells, six photographs of each coverslip were captured with a camera DP73 (Olympus) coupled to an epifluorescence microscope (Olympus BX61). The EtBr fluorescence intensity within the cell was measured with the ImageJ (NIH) software and the average of data from the cells of each experimental condition was calculated to obtain the final measurement of dye uptake in each culture.

### 2.5. Analysis of proliferation in neurospheres

Each experiment was carried out in parallel with two T25 flasks seeded with neurosphere-derived cells from the same culture, at a density of 10,000 viable cells/cm^2^. One of the flasks received drug treatment, and the other (control) was treated with the vehicle. Seventy-two hours after seedling, photographs of the neurospheres were captured using a Leica EC3 camera coupled to a phase-contrast microscope (Leica DMIL-LED) with a 10X objective (eight photographs of random fields per flask). The diameter of the neurospheres was measured with ImageJ. In every experiment, a mean value of the diameter of the neurospheres was obtained for each condition.

The drugs used in our study were: boldine (50 and 100 μM), D4 (inhibitor of connexin hemichannels, 500 nM and 5 μM, Edelris Medicinal Keymistry, Vénissieux, France), and probenecid (inhibitor of Panx1 hemichannels, 0.5 and 1 mM, Sigma Aldrich). Boldine and probenecid were prepared in MilliQ® water, whereas the solvent for D4 solution was ethanol. Concentrated 100X or 1,000X stocks of the drugs were prepared from which the corresponding volumes were added to the flasks receiving treatment. The control flasks received the same volume of the respective vehicle (MilliQ® water or ethanol).

For the analysis of bromodeoxyuridine (BrdU) incorporation, experiments were performed as previously described with the novelty that BrdU (Sigma Aldrich; 1 μM) was added to the flasks for the last 12 h of culture. Then, collected neurospheres from each flask (control and drug treatment) were slightly disaggregated with a P200 pipette and seeded on 12-mm diameter coverslips with DF-12 containing 1% fetal calf serum (Invitrogen), to allow adhesion. After 1 h of seeding on the coverslips, cells were fixed with 70% ethanol (30-min incubation) and processed for BrdU immunohistochemistry, as follows.

BrdU immunohistochemistry was always performed in parallel with coverslips from control and treated cultures of the same experiment. After several washes with PBS, cells in the coverslips were exposed to a DNA denaturation treatment consisting of a 20-min incubation with 2 N HCl. Subsequently, coverlisps were immersed in borate buffer pH 8.5 for 10 min and then washed three additional times with PBS. Then, coverslips were incubated with a solution containing a BrdU antibody (Roche 11170376001; 1:100) for 2.5 h, prepared in 2.5% bovine serum albumin (Sigma-Aldrich) in PBS. After rinsing, coverslips were incubated for 30 min with anti-mouse IgG coupled to FITC (Jackson ImmunoResearch, 1:200), washed several times with PBS and counterstained with DAPI as previously described. After final washes, coverslips were mounted on slides with n-propyl-gallate solution (Sigma-Aldrich, 0.1 M) prepared in glycerol:PBS 9:1.

### 2.6. Analysis of differentiation in neurospheres

Neurospheres were mechanically dissociated and seeded on poly-L-ornithine-treated (Sigma-Aldrich) 12-mm diameter coverslips in DF-12 with 1% fetal calf serum for 4 h to facilitate adhesion, at a density of 10,000 cells/coverslip. Then, coverslips were washed and maintained in DM without growth factor supplementation. Coverslips from the same experiment were divided in two groups: one received 50 μM boldine treatment and the other the corresponding vehicle (MilliQ® water). After 48 h of seeding, neurosphere-derived adhered cells in the coverslips were fixed with 4% paraformaldehyde in 0.1 M phosphate buffer (10-min incubation). Immunohistochemistry was always performed in parallel with coverslips from control and treated cultures of the same experiment. Coverslips were incubated for 30 min in a blocking solution containing 2.5% bovine serum albumin in PBS and then in the primary (2 h at room temperature) and, after rinsing, in the secondary (30 min at room temperature) antibodies prepared in blocking solution. After washing, cells were counterstained with 0.1 μg/mL DAPI for 10 min, washed again and mounted on slides with n-propyl-gallate solution. The primary antibodies used were glial fibrillary acidic protein (GFAP, to identify astrocytes, made in mouse, Sigma Aldrich G3893, 1:1500), chondroitin sulfate proteoglycan (NG2, to identify oligodendrocyte precursors, made in rabbit, Millipore AB320, 1:250), and doublecortin (DCX, to identify neurons, made in goat, Novus Biologicals NBP1-72042, 1:100). The secondary antibodies used were anti-mouse IgG labeled with TRITC, anti-rabbit IgG labeled with FITC, and anti-goat IgG labeled with TRITC (Jackson ImmunoResearch, 1:200).

### 2.7. Epifluorescence microscopy analysis

Coverslips with neurosphere-derived cells from 5 to 8 independent experiments were visualized by epifluorescence microscopy after the immunohistochemical procedures. Fluorescent images of the cells were captured using a camera DP73 (Olympus) coupled to an epifluorescence microscope (Olympus BX61) with a 20X objective. Omission of primary antibodies resulted in the absence of detectable staining in all cases. Six randomly selected fields were captured per coverslip with the excitation wavelengths for the secondary antibody-associated fluorophores (FITC or TRITC) and for DAPI.

Counting of BrdU-positive cells was performed in the images and expressed as percentage of the total number of cells identified by DAPI staining.

Counting of GFAP-, NG2-, or DCX-positive cells was performed on the merged images of the marker with the DAPI channel. Only cells with clear labeling of the cytoplasm or the processes around the DAPI labeled nuclei were considered immunopositive for each marker.

### 2.8. MTT assay

3-(4, 5-dimethylthiazol-2-yl)-2, 5-diphenyltetrazolium bromide (MTT) (Sigma-Aldrich) was used to determine the cell viability and the 50% growth inhibitory concentration (IC_50_) of boldine in GBM59, GBM96, and U87-MG cells. GBM cells were cultured in 24-well plates at a density of 5,000 cells/well in GBM medium. Subsequently, boldine was added to the culture medium at final concentrations of 0, 25, 50, 100, 200, 300, or 600 μM. After 72 h of culture, GBM cells were incubated in the dark with culture medium containing 0.5 mg/mL MTT. After 10 min in dimethyl sulfoxide (DMSO), absorbance was measured at a wavelength of 570 nm using a microplate reader.

### 2.9. Statistics

Statistics were performed with Sigma Plot 11 (Systat Software, San José, CA, United States). All values are expressed as the mean ± standard error of the mean (SEM).

Comparisons between two groups (control and treatment) in experiments with neurospheres or with GBM cells were achieved by using a Student’s *t* test at a significant level of *p* < 0.05.

Comparisons between groups in MTT assay experiments with GBM cells were performed with a one-way ANOVA test at a significant level of *p* < 0.05.

## 3. Results

### 3.1. Boldine inhibits hemichannel activity in neural progenitor cells of the subventricular zone

**Figure 1:**
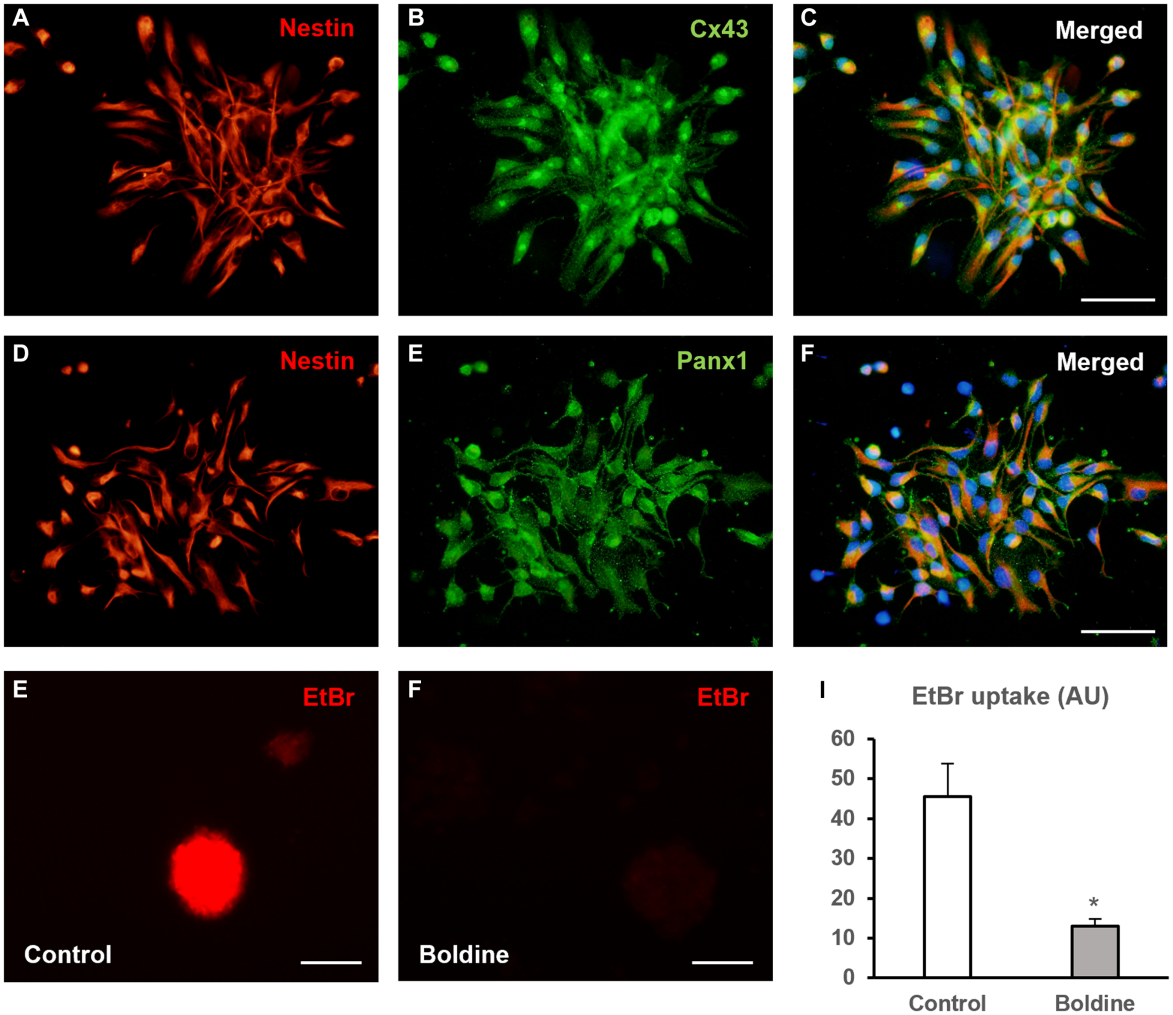
Boldine inhibits hemichannel activity in neural progenitor cells of the postnatal rat subventricular zone. **(A–C)** Epifluorescence microscopy images of neurosphere-derived cells after double immunostaining for nestin [**(A)**, red] and Cx43 [**(B)**, green]. **(C)** shows the merge image of **(A,B)**. Cell nuclei are identified by staining with DAPI (in blue). Scale bar = 50 μm. **(D–F)**. Epifluorescence microscopy images of neurosphere-derived cells after double immunostaining for nestin [**(D)**, red] and Panx1 [**(E)**, green]. **(F)** shows the merge image of **(D,E)**. Cell nuclei are identified by staining with DAPI (in blue). Scale bar = 50 μm. **(G,H)**. Representative fluorescence images of neurospheres incubated in a solution containing 5 μM EtBr for 10 min after a 15-min incubation with milliQ^®^ water **(G)** or with 50 μM boldine **(H)**. Scale bar = 100 μm. The bar chart in **(I)** shows EtBr uptake (in arbitrary units, AU) in cells of neurospheres after 15-min incubation with milliQ^®^ water (control) or with 50 μM boldine. Data are mean ± SEM, *n* = 3, * *p* < 0.05 compared to control condition, *t* test.

In order to demonstrate the presence of hemichannel proteins in NPCs derived from rat postnatal neurospheres we performed immunocytochemical experiments for the detection of Cx43 and Panx1. As seen in Figure 1, most neurosphere-derived NPCs (identified by nestin immunoreactivity) expressed Cx43 (Figures 1A–C) and Panx1 (Figures 1D,E). Boldine, at a concentration of 50 μM, effectively inhibited hemichannel activity in neurosphere-derived cells, as demonstrated by a significant reduction in EtBr uptake (Figures 1G–I, data are mean ± SEM: 45.4 ± 8.4 to 13.0 ± 1.8 arbitrary units, *p* < 0.05, *n* = 3).

### 3.2. Boldine treatment reduces proliferation of neural progenitor cells of the subventricular zone

To analyze whether hemichannel inhibition with boldine affects NPC proliferation, we measured the diameter of the neurospheres formed after 72 h of incubation of NPCs with vehicle (control) or with 50 μM boldine. Additionally, we compared the percentage of BrdU incorporation during the last 12 h of culture between control and boldine treatment conditions.

**Figure 2:**
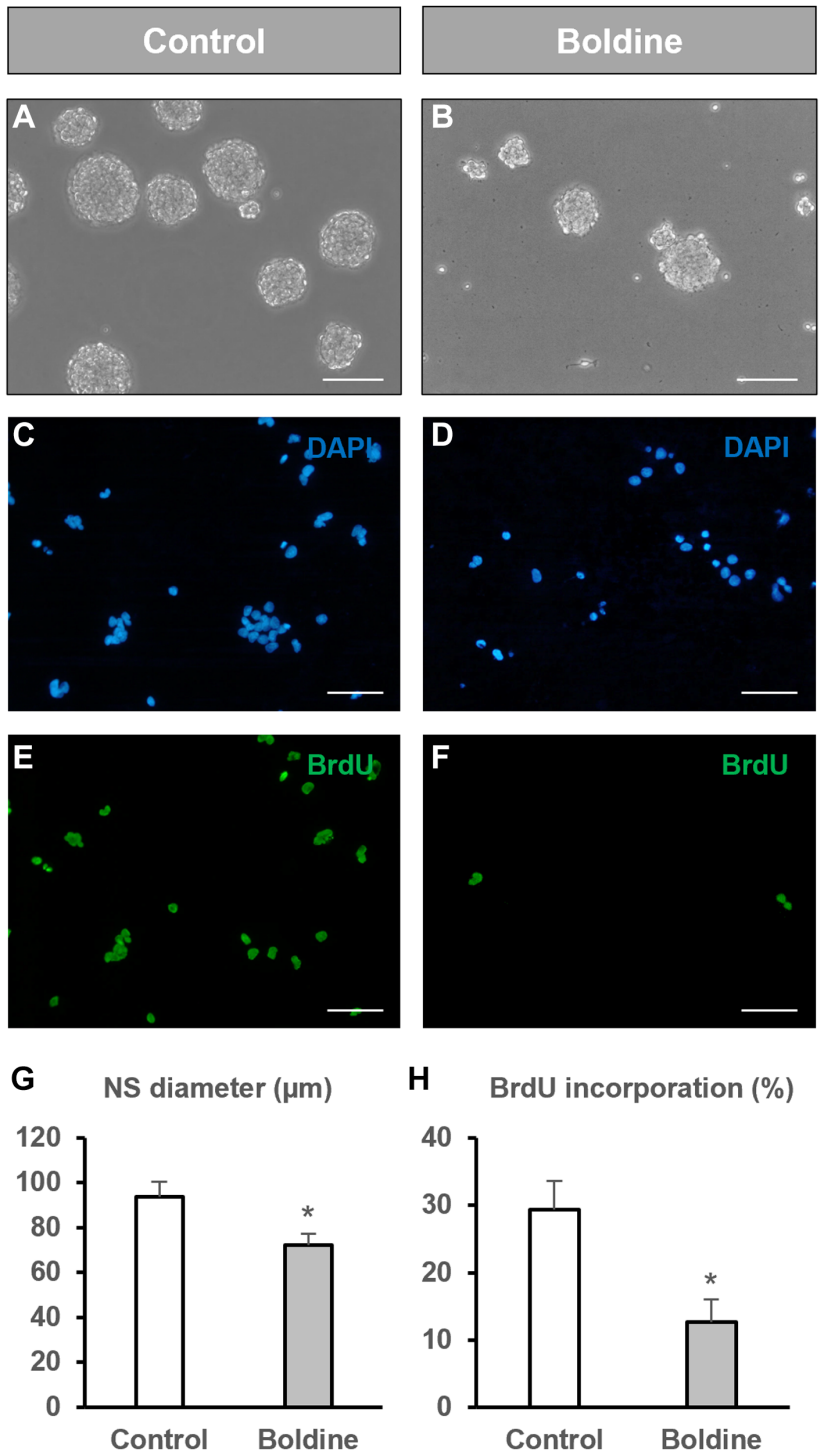
Effect of boldine treatment on neurosphere diameter and BrdU incorporation. Neural progenitor cells (NPCs) of the postnatal rat subventricular zone were cultured in the absence (control) or presence of the hemichannel blocker boldine. The diameter of the formed neurospheres 72 h after seeding, as well as the percentage of bromodeoxyuridine (BrdU) incorporation during the last 12 h of culture were evaluated. **(A,B)**: Phase-contrast photomicrographs of neurospheres in control cultures **(A)** and in cultures treated with 50 μM boldine **(B)**. Scale bar: 100 μm. **(C–F)**: Epifluorescence images showing BrdU immunohistochemistry (in green) in neurosphere-derived cells obtained from control cultures and cultures treated with 50 μM boldine. The total number of cells in each field was identified by DAPI staining (in blue). Scale bar: 50 μm. **(G)**: Graph showing the neurosphere (NS) diameter (in μm) in each experimental condition. Data are the mean ± SEM, *n* = 4, * *p* < 0.05, *t* test. **(H)**: Graph showing the percentage of BrdU incorporation in both experimental conditions. Data are the mean ± SEM, *n* = 8, * *p* < 0.05, *t* test.

Neurosphere diameter was significantly reduced by 23% after boldine treatment (93.6 ± 6.7 μm in control cultures to 72.15 ± 5.2 μm in boldine-treated cultures, *p* < 0.05, *n* = 4) (Figures 2A,B,G). We also tested the effect of an increased boldine concentration, 100 μM, on neurosphere diameter. Treatment of NPCs with 100 μM boldine affected neurosphere integrity leading to the production of opaque neurospheres and multiple individual, nonforming neurosphere cells (Supplementary Figure 1). Therefore, the effect of boldine treatment on BrdU incorporation was tested only with the 50 μM concentration. The percentage of BrdU incorporation in neurosphere-derived cells after 72 h treatment with 50 μM boldine was significantly reduced from a 29.3 ± 4.3% to a 12.7 ± 3.3% (*p* < 0.05, *n* = 8) (Figures 2C–F,H).

**Figure 3:**
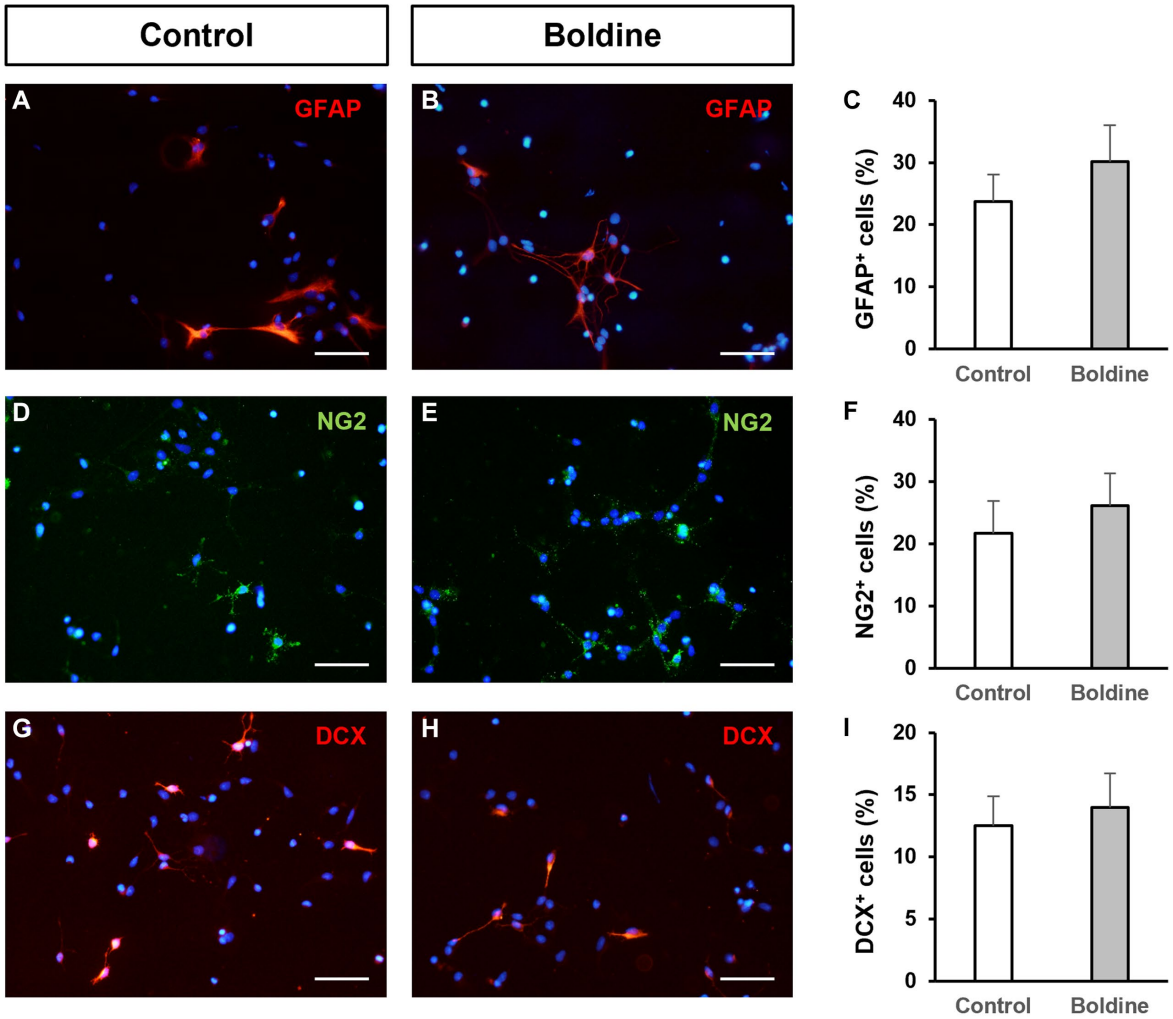
Effect of boldine treatment on neural progenitor cell differentiation. Fluorescence microscopy images of neurosphere-derived adhered cells immunostained with antibodies to identify astrocytes [GFAP, red, **(A,B)**], oligodendroglial precursors [NG2, green, **(D,E)**], and neurons [DCX, red, **(G,H)**], and counterstained with DAPI (blue), in cultures treated for 48 h with vehicle [control, **(A,D,G)**] or with 50 μM boldine **(B,E,H)**. Scale bars: 50 μm. **(C,F,I)**: Percentage of cells immunoreactive to GFAP **(C)**, NG2 **(F)**, and DCX **(I)**, in neurosphere-derived cells grown on adhesive substrate for 48 h and treated either with vehicle (control) or with 50 μM boldine. Data are the mean ± SEM, *n* = 8, *p* > 0.05, *t* test.

We also aimed to test putative effects of boldine treatment on NPC differentiation. For that purpose, we seeded NPCs on differentiation-inducing conditions, that is, on an adhesive substrate and in the absence of growth factors. After 48 h of culture under these conditions, 23.7 ± 4.3% of the seeded NPCs differentiated to astrocytes (Figures 3A,C), 21.7 ± 5.1% to oligodendrocyte-progenitor cells (Figures 3D,F), and 12.5 ± 2.3% to neurons (Figures 3G,H). Treatment of the cultures with 50 μM boldine for 48 h did not induce any significant modification in this differentiation pattern (30.1 ± 5.8% astrocytes (Figures 3B,C), 26.1 ± 5.2% oligodendrocyte-progenitor cells (Figures 3E,F), and 14.0 ± 2.7% neurons (Figures 3H,I), *p* > 0.05, *n* = 8).

### 3.3. Other hemichannel blockers differentially affect neural progenitor cell proliferation

**Figure 4:**
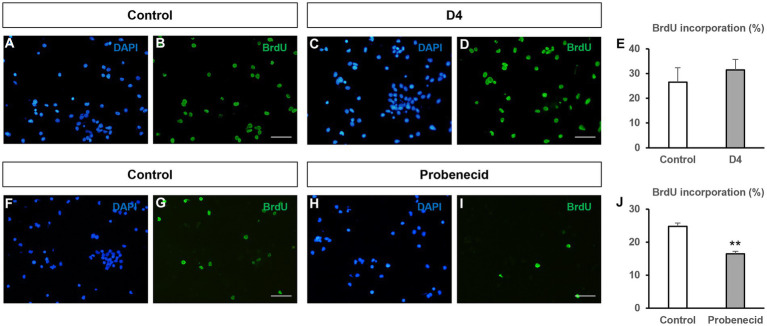
Effect of other hemichannel blockers on BrdU incorporation in neural progenitor cells. Neural progenitor cells (NPCs) of the postnatal rat subventricular zone were cultured for 72 h in the absence (control) or presence of the connexin hemichannel blocker D4 (500 nM) or the Panx1 hemichannel blocker probenecid (1 mM). The percentage of bromodeoxyuridine (BrdU) incorporation during the last 12 h of culture was evaluated. **(A–D)**: Epifluorescence images showing BrdU immunohistochemistry (in green) in neurosphere-derived cells obtained from control cultures and cultures treated with 500 nM D4. The total number of cells in each field was identified by DAPI staining (in blue). Scale bar: 50 μm. **(E)**: Graph showing the percentage of BrdU incorporation in both experimental conditions. Data are the mean ± SEM, *n* = 8, *p* > 0.05, *t* test. **(F–I)**: Epifluorescence images showing BrdU immunohistochemistry (in green) in neurosphere-derived cells obtained from control cultures and cultures treated with 1 mM probenecid. The total number of cells in each field was identified by DAPI staining (in blue). Scale bar: 50 μm. **(J)**: Graph showing the percentage of BrdU incorporation in both experimental conditions. Data are the mean ± SEM, *n* = 6, ** *p* < 0.001, *t* test.

Since hemichannels blocked by boldine can be formed by either connexin or pannexin proteins, we decided to test the effect on NPC proliferation of other drugs that specifically target hemichannels formed specifically by connexins or pannexins. Therefore, we first analyzed the effect of D4, a molecule that inhibits connexin hemichannels, but not connexin gap junctions (Cisterna et al., 2020). As seen in Figures 4A–E, D4 (500 nM) treatment of neurosphere-derived cells did not significantly affect BrdU incorporation (26.5 ± 5.8% in control cultures *vs* 31.4 ± 4.2% in cultures treated with 500 nM D4, *n* = 8, *p* > 0.05), which suggests that Cx43 hemichannel inhibition is not relevant for NPC proliferation. A 10X increase in D4 concentration (5 μM) did not modify BrdU incorporation in NPCs either (data not shown). Next, we proceeded to treat NPC cultures with probenecid, a molecule that inhibits Panx1 hemichannels (Silverman et al., 2008). Previous findings demonstrated that 1 mM probenecid reduced neurosphere diameter in NPC cultures after 7 days *in vitro* (Wicki-Stordeur et al., 2012). Therefore, we decided to use this concentration to evaluate BrdU incorporation under our experimental conditions. Interestingly, BrdU incorporation in NPCs treated with 1 mM probenecid resulted significantly reduced as compared to NPCs treated with the vehicle (Figures 4F–J; 24.8 ± 1.0% in control cultures *vs* 16.4 ± 0.8% in cultures treated with probenecid, *n* = 6, *p* < 0.001).

### 3.4. Boldine inhibits cell growth in glioblastoma cells

**Figure 5:**
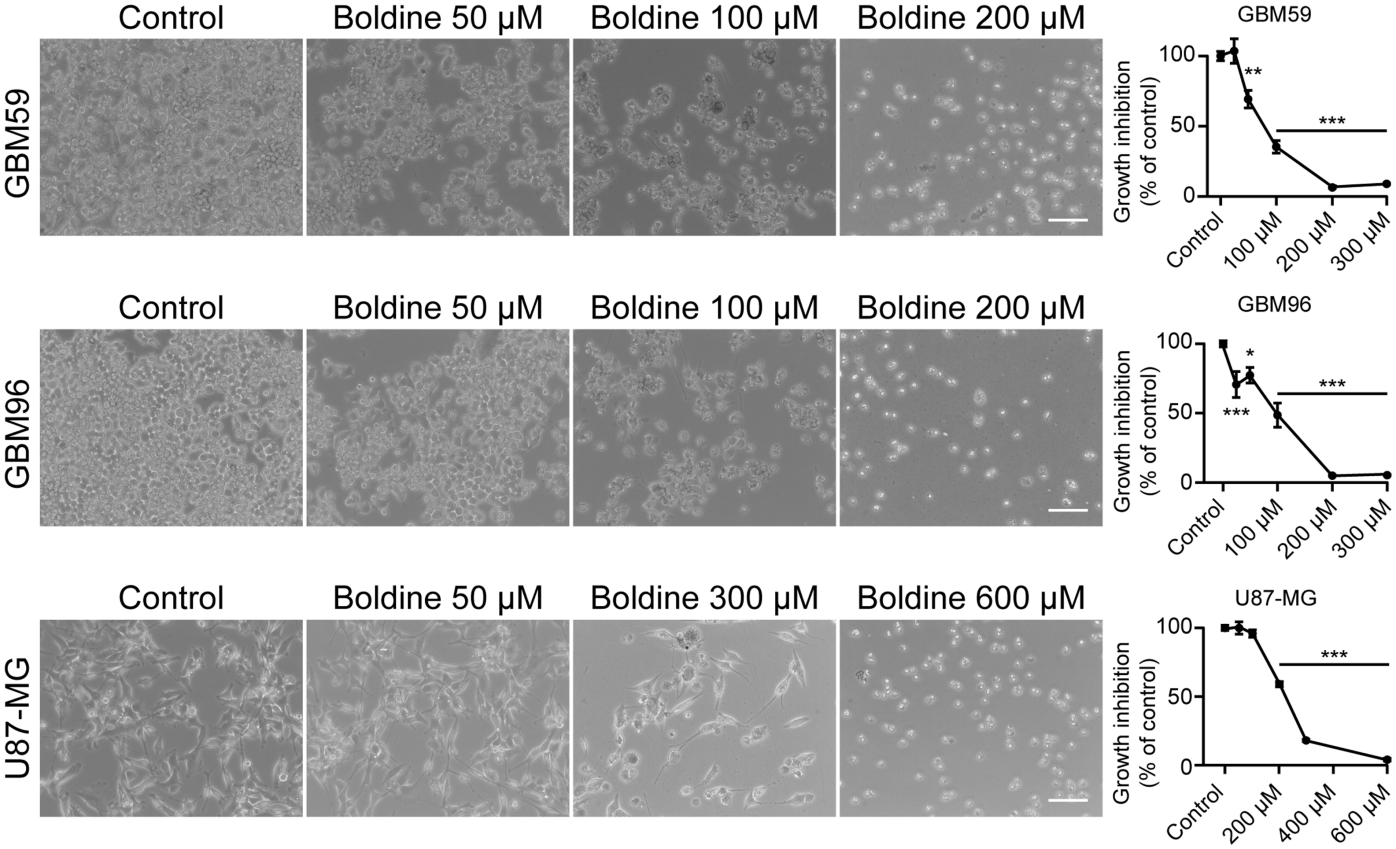
Effect of boldine on glioblastoma cell growth. Cell viability in GBM cells treated for 72 h with different concentrations of boldine (25, 50, 100, 200, and 300 μM for GBM59 and GBM96 cells, and 50, 100, 200, 300, and 600 μM for U87-MG cells). Phase-contrast photomicrographs in the left panel show examples of GBM59, GBM96, and U87-MG cells in control conditions and after 72 h of treatment with boldine at the indicated concentrations. Scale bars: 100 µm. Bar histograms in the right panel show the growth inhibition (as percentage with respect to control) of every tested concentration of boldine in GBM59, GBM96 and U87-MG cells. Data are the mean ± SEM, *n* = 9–12, *p < 0.05, **p < 0.001, ***p < 0.0001, compared to control, one-way ANOVA test.

Based on common features between SVZ NPCs and glioma stem cells we proceeded to analyze the effect of boldine on cell growth in three different primary human GBM cells: GBM59, GBM96, and U87-MG, using the MTT assay. Five boldine concentrations were tested to perform concentration-dependent curves and to calculate the IC_50_ values. As seen in Figure 5, boldine induced a concentration-dependent reduction in cell growth in the three tested GBM cell lines. U87-MG cells were less sensitive to boldine treatment than GBM59 and GBM96 cells. Therefore, higher boldine concentrations were required to achieve significant reductions in cell growth (Figure 5). The IC_50_ values obtained were: 68.6 μM, 141.7 μM, and 213.8 μM, for GBM59, GBM96, and U87-MG cells, respectively.

### 3.5. Boldine reduces EtBr uptake in glioblastoma cells

To analyze whether boldine treatment affects hemichannel activity in GBM cells we first demonstrated the presence of hemichannel proteins in these cells. As seen in Supplementary Figure 2, both Cx43 and Panx1 were expressed by the three tested GBM cell lines.

**Figure 6:**
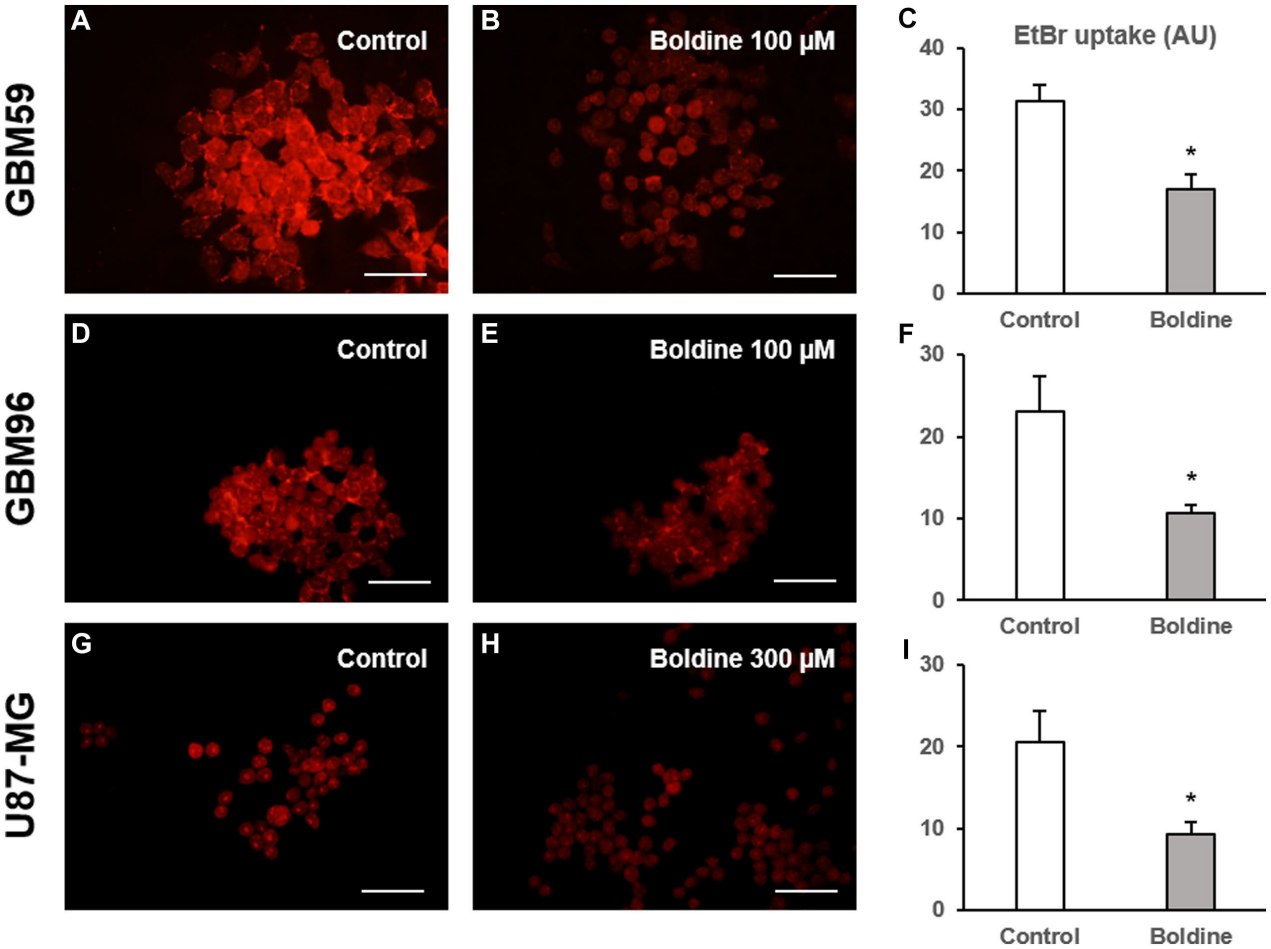
Boldine reduces hemichannel activity in glioblastoma cells. Representative fluorescence images of GBM59 **(A,B)**, GBM96 **(D,E)**, and U87-MG cells **(G,H)** incubated in a solution containing 5 μM EtBr (in red) for 10 min after a 15-min incubation with milliQ^®^ water (control) or with boldine at the indicated concentrations. Scale bars: 100 μm. **(C,F,I)**: Bar charts showing EtBr uptake (in arbitrary units, AU) in GBM59, GBM96, and U87-MG cells after 15-min incubation with milliQ^®^ water (control) or with boldine (100 μM for GBM59 and GBM96 cells, and 300 μM for U87-MG cells). Data are mean ± SEM from 3 to 4 independent experiments, * *p* < 0.05 compared to control condition, *t* test.

Subsequently, EtBr uptake was evaluated in GBM cells in the absence or presence of boldine. As seen in Figure 6, boldine, at concentrations close to the IC_50_ of each GBM cell line, produced a significant reduction in EtBr uptake in GBM59, GBM96 and U87-MG cells. These results demonstrate that boldine inhibits hemichannel activity in patient-derived GBM cells.

## 4. Discussion

In this article, we show that boldine, a molecule that inhibits hemichannels, but not gap junctions, exerts an antiproliferative effect on postnatal rat SVZ NPCs which indicates that, communication mediated by hemichannels intervenes in the control of NPC proliferation. Boldine treatment also induces a significant reduction in GBM cell growth. Therefore, the alkaloid boldine might be an interesting compound to test, alone or in combination with temozolomide, as an adjuvant therapy for gliomas or to reduce the possibility of recurrences after tumor resection.

Boldine has been extensively reported as a potent natural antioxidant and possesses several health-promoting properties mainly based on its free radical scavenger and/or anti-inflammatory actions (Backhouse et al., 1994; O’Brien et al., 2006). In addition to these well-known effects, boldine has been reported to inhibit hemichannel activity without affecting gap junctional communication (Hernández-Salinas et al., 2013; Yi et al., 2017) and to block P2X_7_ receptors (Cea et al., 2023; Toro et al., 2023). The inhibition of these channels and receptors might be involved in boldine-induced antioxidant effects since, as Ca^2+^ promotes the formation of superoxide anions, hemichannel blockers might prevent the generation of free radicals by blocking the influx of Ca^2+^ (Balboa et al., 2020). In fact, this mechanism of action accounts for additional protective effects, such as decreased neuronal damage in a murine model of Alzheimer’s disease (Yi et al., 2017) and myofiber protection in a mouse model of muscular dystrophy (Cea et al., 2020) or after endotoxemia (Cea et al., 2019).

In this work, we have demonstrated the presence of hemichannel proteins (Cx43 and Panx1) in NPCs of the SVZ grown as neurospheres. In a previous article we showed that neurosphere-derived NPCs express mRNA for different hemichannel protein subtypes and present hemichannel activity in control conditions (Talaverón et al., 2015). Other authors have also reported the existence of Cx43 and Panx1 in the NPC population in the SVZ neurogenic niche (Wicki-Stordeur et al., 2012; Ravella et al., 2015) and they have defined possible roles of gap junctional communication between NPCs within the SVZ (Lacar et al., 2011; Ravella et al., 2015). When used for therapeutic purposes, NPCs are also able to form functional gap junctions with the host tissue that could be relevant for their survival and beneficial effects (Jäderstad et al., 2010; Talaverón et al., 2014). However, the effect of the communication by hemichannels in this population of progenitor cells has not been analyzed yet. By selectively inhibiting hemichannels, but not gap junctions, with boldine, we have shown that the proliferation rate of cultured SVZ NPCs is reduced by 55%. Interestingly, NPC differentiation pattern was not affected by boldine treatment which suggests that the entry or the release of molecules by hemichannels might intervene in the control of the mitotic activity of NPCs but not in their commitment to a specific lineage.

As boldine inhibits hemichannels formed both by connexins or pannexins, to elucidate possible involvements of specific protein subtypes, we analyzed BrdU incorporation in NPCs treated with a selective connexin hemichannel blocker (D4) or a Panx1 hemichannel blocker (probenecid). These experiments revealed that communication mediated by Cx43 hemichannels did not exert a significant role in NPC proliferation. Interestingly, Panx1 channel inhibition reduced NPC proliferation, suggesting that, among the different hemichannel subtypes present in NPCs, at least those formed by Panx1 might have a role in controlling NPC division. This agrees with a previous study by Wicki-Stordeur et al. (2012) showing a reduction in neurosphere diameter in cultures of neonatal mouse SVZ NPCs treated with 1 mM probenecid. ATP is most likely the molecule involved in actions derived from Panx1-mediated communication. The interaction of ATP with different purinergic receptors (mainly P2X7 and P2Y1) modulates adult neurogenesis. Activation of P2Y1 receptors leads to increased proliferation and migration, and this effect is counterbalanced by P2X7 activation that decreases proliferation, induces neuronal differentiation, and apoptosis (Cavaliere et al., 2015). Whether ATP is a molecule involved in boldine antiproliferative actions in SVZ NPCs remains to be elucidated.

Connexins and pannexins can also interact through their cytoplasmic domains with different molecules to induce relevant effects on cell growth, migration, or differentiation (Leithe et al., 2018; Laird and Penuela, 2021). Hemichannel blockers may exert additional effects on the protein channel interactome, and vice versa. For example, probenecid has been described to disrupt the interaction of Panx1 with the microtubule-associated protein Crmp2, leading to an increase in microtubule stability (Xu et al., 2018). The penetrating peptide TAT-Cx43_266-283_, which mimics Cx43 interactions with the nonreceptor tyrosine kinase c-Src, also produces hemichannel inhibition (Gangoso et al., 2017). Therefore, we cannot rule out the possibility that, through hemichannel inhibition-independent actions, boldine may interfere with the connexin/pannexin interactome leading to antimitotic effects.

Recent evidence supports the notion that primary *IDH*-wild type GBM may arise from SVZ NSCs that acquire driver mutations (Lee et al., 2018). Furthermore, glioma stem cells have many similarities with SVZ NSCs, such as nestin expression, high motility, diversity of progeny, proliferative potential, association with blood vessels, and bilateral communication with constituents of the niche (Sanai et al., 2005). In our study, we have demonstrated that boldine causes a significant reduction in cell growth in different types of primary GBM cells. Furthermore, we have shown for the first time that boldine inhibits hemichannel activity in GBM cells. Other authors reported earlier that boldine treatment is effective in reducing the mitotic index of other human and rat glioma cell lines, without affecting apoptosis or being toxic to normal brain cells (Gerhardt et al., 2009). Treatment with boldine has also been shown to reduce the proliferation of other tumor cells such, as breast cancer cells, *in vitro* and *in vivo* (Paydar et al., 2014), although the specific mechanisms by which boldine exerts these antimitotic actions have not been detailed. Additional studies will be necessary to be performed to unravel whether the antiproliferative effect of boldine in GBM cells is derived from the interruption of ion/molecular transfer across open hemichannels or from hemichannel-independent effects.

Our results showing similar antiproliferative actions of boldine in SVZ NPCs and in primary GBM cells could be of clinical relevance to use this drug as a potential therapy for GBM, alone or in combination with existing chemotherapy, to reduce both the proliferation of GBM cells and SVZ NSCs that could act as tumor-initiating cells.

## Data availability statement

The raw data supporting the conclusions of this article will be made available by the authors, without undue reservation.

## Ethics statement

The animal study was reviewed and approved by Comité Ético de Experimentación Animal (Animal Research Ethics Comittee), University of Seville, Spain.

## Author contributions

EM, JS, AP, and AT designed the study and analyzed the data. EJ-M, CM-D, RT, and EM performed the experiments. EM wrote the first draft of the manuscript. All authors contributed to manuscript revision, read, and approved the submitted version.

## Funding

This publication was supported by project P20_00529 (Consejería de Economía, Conocimiento, Empresas y Universidad, Junta de Andalucía-FEDER). Research was also funded by project SA125P20 (Consejería de Educación, Junta de Castilla y León, FEDER). We also acknowledge funding from VII Plan Propio de Investigación y Transferencia, University of Seville.

## Conflict of interest

The authors declare that the research was conducted in the absence of any commercial or financial relationships that could be construed as a potential conflict of interest.

## Publisher’s note

All claims expressed in this article are solely those of the authors and do not necessarily represent those of their affiliated organizations, or those of the publisher, the editors and the reviewers. Any product that may be evaluated in this article, or claim that may be made by its manufacturer, is not guaranteed or endorsed by the publisher.
