# Supplementary material for: Antiproliferative effect of boldine on neural progenitor cells and on glioblastoma cells
Source: Front Neurosci. 2023 Aug 16;17:1211467. doi: 10.3389/fnins.2023.1211467 (PMC10467274; doi:10.3389/fnins.2023.1211467)
Supplement: Supplementary file 1 [file Data_Sheet_1.PDF]

Supplementary Material

Antiproliferative effect of boldine on neural progenitor cells and on glioblastoma cells

Enrique Jiménez-Madrona[1,2,#], Camilo J. Morado-Díaz[1#], Rocío Talaverón[2, 3], Arantxa Tabernero[2], Ángel M. Pastor[1], Juan C. Sáez[4] and Esperanza R. Matarredona[1*]

[1] Departamento de Fisiología, Facultad de Biología, Universidad de Sevilla, Seville, Spain

[2]Instituto de Neurociencias de Castilla y León (INCYL), Universidad de Salamanca, Salamanca, Spain

[3]Departamento de Bioquímica y Biología Molecular, Facultad de Farmacia, Universidad de Sevilla, Seville, Spain

[4] Instituto de Neurociencia, Centro Interdisciplinario de Neurociencias de Valparaíso, Facultad de Ciencias, Universidad de Valparaíso, Valparaíso, Chile.

# contributed equally to this report

*** Correspondence:**
Esperanza R. Matarredona
matarredona@us.es

Supplementary Figures

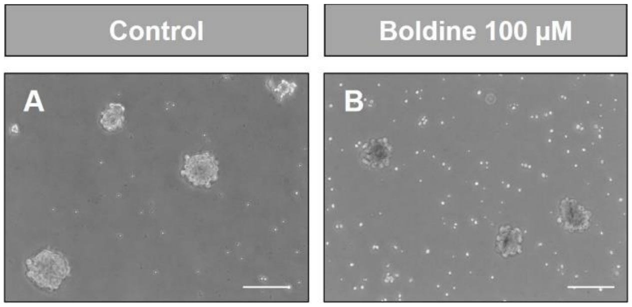

Supplementary Figure 1: Phase-contrast photomicrographs of neurospheres in control cultures (A) and in cultures treated with 100 µM boldine (B). Scale bar: 100 µm.

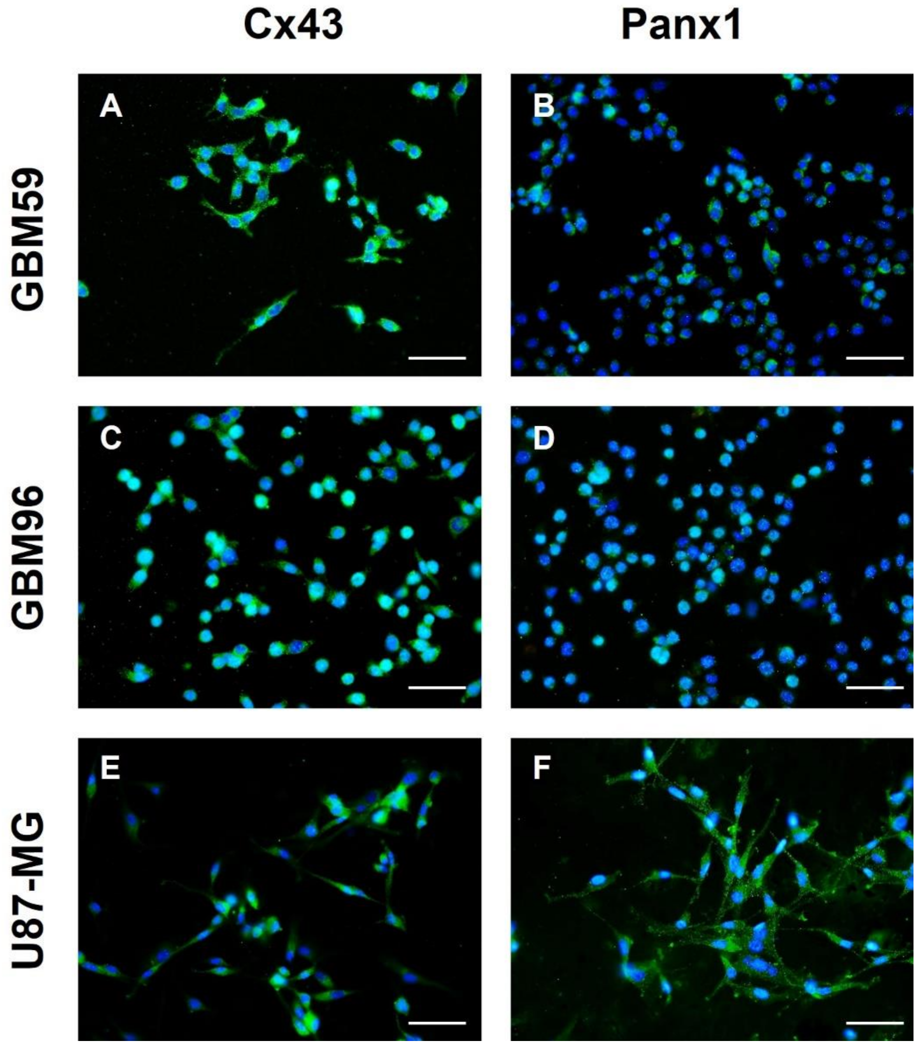

Supplementary Figure 2. Cx43 and Panx1 expression in glioblastoma cell lines. Epifluorescence microscopy images of glioblastoma cells (GBM59 in A and B, GBM96 in C and D, U87-MG in E and F) after immunostaining for the detection of Cx43 (green, A, C, E) or Panx 1 (green, B, D, and F). Cell nuclei are identified by staining with DAPI (in blue). Scale bars = 50 µm.

.
